# Benefits of Group Foraging Depend on Prey Type in a Small Marine Predator, the Little Penguin

**DOI:** 10.1371/journal.pone.0144297

**Published:** 2015-12-16

**Authors:** Grace J. Sutton, Andrew J. Hoskins, John P. Y. Arnould

**Affiliations:** 1 Deakin University, School of Life and Environmental Sciences (Burwood Campus), Geelong, Australia; 2 CSIRO Land and Water, Canberra, Australian Capital Territory, Australia; UC Santa Cruz Department of Ecology and Evolutionary Biology, UNITED STATES

## Abstract

Group foraging provides predators with advantages in over-powering prey larger than themselves or in aggregating small prey for efficient exploitation. For group-living predatory species, cooperative hunting strategies provide inclusive fitness benefits. However, for colonial-breeding predators, the benefit pay-offs of group foraging are less clear due to the potential for intra-specific competition. We used animal-borne cameras to determine the prey types, hunting strategies, and success of little penguins (*Eudyptula minor*), a small, colonial breeding air-breathing marine predator that has recently been shown to display extensive at-sea foraging associations with conspecifics. Regardless of prey type, little penguins had a higher probability of associating with conspecifics when hunting prey that were aggregated than when prey were solitary. In addition, success was greater when individuals hunted schooling rather than solitary prey. Surprisingly, however, success on schooling prey was similar or greater when individuals hunted on their own than when with conspecifics. These findings suggest individuals may be trading-off the energetic gains of solitary hunting for an increased probability of detecting prey within a spatially and temporally variable prey field by associating with conspecifics.

## Introduction

In spatially and temporally dynamic environments, animals must adapt their foraging strategies in order to optimise their efforts and to reduce metabolic costs [1]. A predator’s hunting strategy will be greatly influenced by the size of its prey relative to itself [2]. Individuals may employ solitary hunting strategies when the costs of searching, catching and handling prey are lower than the prey’s energetic rewards whereas group hunting should be beneficial for coordinating efforts to subdue large prey or creating concentrated aggregations of small prey for efficient exploitation [3]. For social predatory species, cooperative group hunting strategies provide inclusive fitness benefits [4]. However, for colonial-breeding predators, the benefit pay-offs of group foraging are less clear due to the potential for intra-specific competition.

In order to increase foraging success, predators must constantly seek out locations with a high abundance and quality of prey, whereas prey should seek areas with lower densities of predators [5]. As prey is distributed unpredictably in the environment, animals may gain insight into location of patches by detecting conspicuous cues provided from other foraging individuals [1]. This opportunistic foraging strategy, known as *local enhancement*, is thought to reduce search effort [5]. However it may not benefit individuals if prey is not particularly abundant and competition to consume the prey ensues once it is detected [6].

The energetic density of certain prey types can provide important fitness advantages resulting in some foraging behaviours being more successful than others. Correspondingly, a predator’s foraging decisions may reflect the type and abundance of prey available [6]. Previous studies have predicted animals should adjust their foraging strategies in relation to how prey is dispersed in the environment [1]. However, such variability in strategies is rarely observed in free-ranging predators. This is especially so for marine vertebrates due to the difficulties of observing their behaviour at sea [7].

Central place foraging predators such as seabirds face spatial constraints during the breeding season due to the need to regularly return to the nest to provision offspring [8]. During this time it is vital for individuals to employ strategies which reduce the energetic cost of searching for prey and travel in times when it is particularly important to optimise foraging performance. Recent studies have shown breeding little penguins (*Eudyptula minor*) spend considerable time at sea foraging in groups [9] which is commensurate with a diet of small schooling fish. Numerous studies have shown the species also consumes solitary prey and krill [10] which should elicit different foraging behaviours relative to the nutritional pay-off of the prey. Whether little penguins adapt their hunting strategies in response to prey characteristics (schooling *versus* solitary, small *versus* large) is not known.

The aims of the present study, therefore, were to determine: 1) if hunting strategies (specifically, group foraging *versus* solitary foraging) are influenced by prey type; and 2) whether this affects individual foraging success and energy gain.

## Materials and Methods

### Ethics Statement

The present study was conducted in accordance with the guidelines of Deakin University Animal Ethics Committee (Approval B21-2013) and under a Wildlife Research Permit (10006877) from the Department of Environment and Primary Industries (Victoria). London Bridge in Port Campbell National Park and Gabo Island Lighthouse Reserve were accessed under permit from Parks Victoria.

### Animal handling and instrumentation

In order to increase the likelihood of detecting different prey types encountered by little penguins, the study was conducted at two breeding colonies in south-eastern Australia: London Bridge (LB, 38°62’S, 142° 93’E) and Gabo Island (GI, 37°56’S, 149°91’E). During the 2014–15 (October-January) breeding period, adults in early chick rearing stage were captured in their nest burrows before sunrise, weighed in a cloth bag using a spring balance (± 10 g) and measured using Vernier callipers (± 1 mm) to determine sex [11]. Individuals were instrumented with video data loggers (Catnip Technologies Ltd., U.S.A, 30 x 40 x 15 mm, 20 g, 400 x 400 pixels at 30 frames·s^−^¹) programmed to record on a duty cycle of 15 minutes every hour. In addition, individuals were instrumented with a GPS (IgotU120, Mobile Action Technology, 44.5 x 28.5 x 13 mm, 12 g in air) and a time-depth recorder (TDR LAT 1500, Lotek Wireless Inc., 35 x 8 x 8 mm, 3.4 g in air). The device package was attached along the dorsal midline using Tesa^®^ tape (4651), with the camera facing forward in front of the GPS-TDR units. Together, the devices weighed <3% of the average body mass and was <1% cross sectional surface area of little penguins. Birds were then released into their burrow and devices were removed after a single foraging trip.

### Data processing and statistical analyses

All data analyses was conducted in the R Statistical Environment [12]. Dive logger data were corrected for depth drift and summarised using the package *diveMove* and the *trip* package was used to summarise GPS data following the application of a speed filter [13]. Video data were analysed and successful prey patch encounters, defined as instances when prey were visible in the footage and captured in the presence or absence of conspecifics, were recorded. Dives where individuals did not encounter prey were not included in the analysis. Prey were categorised as aggregated or solitary and were identified to the lowest taxonomic level possible with aid of a fish identification guide [14]. An index of foraging success was calculated as the total number of prey caught per dive at each prey patch. Finally, an index of foraging energy gain was calculated as the total gross energy (GE; kJ) consumed per dive (Table 1).

**Table 1:** Estimated energy content of prey observed to be consumed by little penguins (*Eudyptula minor)* in videos.

Prey Species	Energy content (kJ·g^-1^ WM)	Mean mass (g)	Total GE (kJ)	Reference
Coastal krill	3	0.02	0.06	[15, 16]
(*Nyctiphanes australis*)				
Southern anchovy	5.2	5.5	28.6	[17, 18]
(*Engraulis australis*)				
Sandy sprat (*Hyperlophus vittatus*)	5	2.6	13	[18]
*Cyanea spp*	0.4	2.88	1.2	[19]
Juvenile *Clupeiformes*	2.2	0.92	2	[18]

The prey capture data recorded using the video loggers was hierarchically nested with multiple foraging dives recorded per-animal and individuals sampled from two separate colonies. To account for this non-independence of samples, all models were fitted using a mixed-effects modelling framework [20, 21]. Random terms were included in all models that consisted of individual bird ID nested inside of colony of origin.

The comparison between the presence of conspecifics and prey type was modelled using a Generalised Linear Mixed-effects Model (GLMM). A Bernoulli response variable representing the presence (1) or absence (0) of conspecifics was fitted against a 9 category predictor variable with individual categories representing both prey type and if the prey were aggregated or solitary. Due to the binary nature of the data, the model used a binomial error structure with logistic link function to provide estimates of the likelihood of conspecific association for each predictor prey category. The model was fitted using the *lme4* package in R (v 3.2.1;[22]).

Linear Mixed-Effects models (LME) were used to compare the responses of prey capture success and the estimated total gross energy per dive to the same 9 category predictor for prey type used in the GLMM described above and a second two-level categorical predictor identifying the presence of conspecifics. Model selection was undertaken to identify the most parsimonious model from the full model containing two categorical predictors and their interaction using Akakie’s Information Criterion [23]. When needed, heteroscedasticity in residual spread was accounted for using an exponential variance function. Models were fitted using the *nlme* package (v 3.1–120; [24]).

Unless otherwise indicated, data are presented as Mean ± Standard Error.

## Results

A total of 21 individuals were instrumented (10 at LB; 11 at GI). Foraging trip duration (17.6 ± 1.8 h), mean dive depth (8.2 ± 0.1 m) and total distance travelled (50.3 ± 6.8 km, S1 Appendix) were all within the range previously reported for little penguins at the two study sites [9] and, therefore, it was assumed that individuals were not affected by the slightly larger devices used during the current study [25]

Due to device malfunctions, not all video cameras recorded at the programmed duty cycle. Nonetheless, video data were obtained at intervals dispersed throughout the whole foraging trip for an average combined duration of 3.5 ± 0.3 h (range 1.1–5.5 h, S1 Appendix).

**Fig 1:**
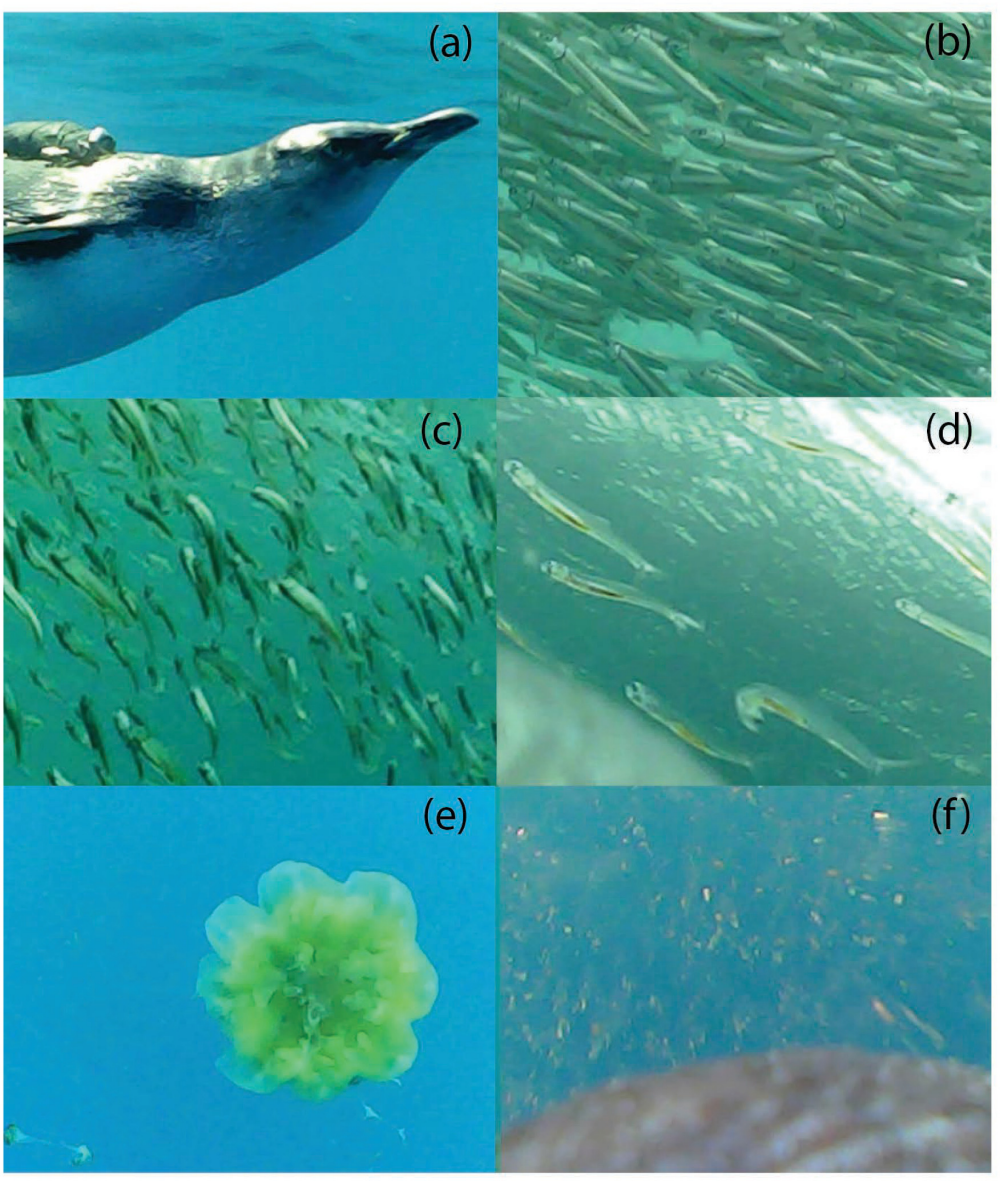
Representative images of the types of little penguin foraging data obtained during this study: (a) conspecific showing position of camera package; (b) southern anchovy, (c) sandy sprat, (d) juvenile *Clupeiformes*, (e) Cyaneidae jellyfish and (f) coastal krill.

A total of 295 prey encounters were observed representing 5 main prey types (southern anchovy, *Engraulis australis*; sandy sprat, *Hyperlophus vittatus*; coastal krill, *Nyctiphanes australis*; juvenile *Clupeiformes*; and jellyfish, *Cyanea spp*; Fig 1). Four solitary large fish (three *Perciformes spp*. and one *Sygnathidae sp*.) were observed to be consumed but these were excluded from further analyses due to their infrequency. Individuals encountered 14.9 ± 4.3 prey patches (4.2 ± 0.1 prey patches·h^-1^ of video data) where they hunted alone or with conspecifics. The maximum number of conspecifics observed at any one time was 24 and 4 at GI and LB, respectively.

Prey were consumed at depth immediately after capture (S1 Video). When hunting schooling prey, individuals consumed >1 prey during 53% of dives (mode = 2 prey per dive). However, smaller prey (juvenile *Clupeiformes* and krill) were consumed at greater rates (Table 2). Prey handling time was < 2 s per prey item except for jellyfish which took up to 35 s as individuals broke apart these large prey.

**Table 2:** Summary of all prey capture events observed from animal-borne cameras on little penguins.

Prey type	Schooling/solitary	Prey events (*n*)	
		Conspecifics present	Conspecifics absent
Southern anchovy	Schooling	10	12
Southern anchovy	Solitary	6	58
Coastal krill	Schooling	1	2
Sandy sprat	Schooling	6	6
Sandy sprat	Solitary	1	12
*Cyanea* spp	Schooling	1	8
*Cyanea* spp	Solitary	5	70
Juvenile *Clupeiformes*	Schooling	11	31
Juvenile *Clupeiformes*	Solitary	4	50
Total		45	250

**Fig 2:**
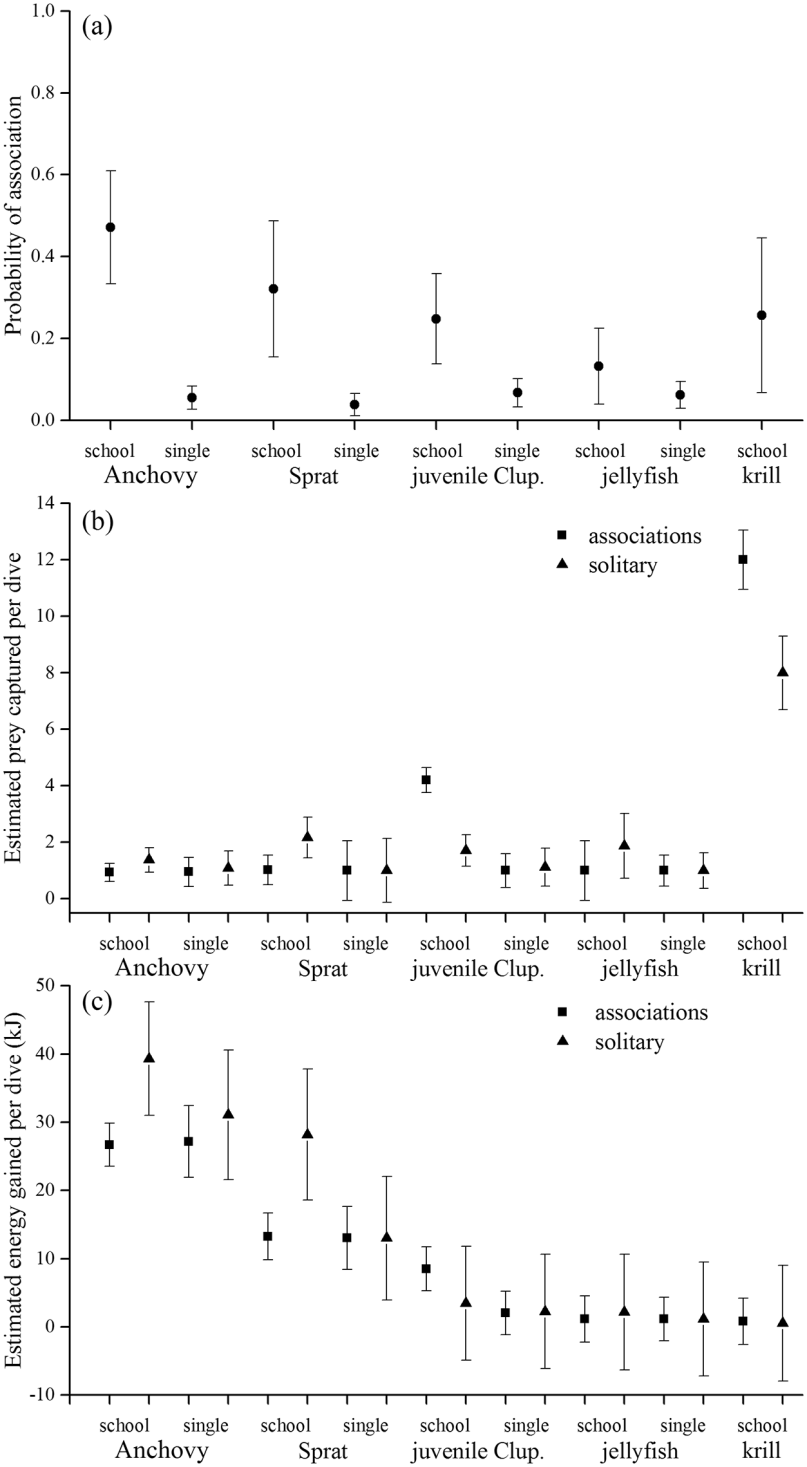
Results of mixed models: Probability of little penguins associating with conspecifics (2a), number of prey consumed per dive (2b) and energy gain per dive (2c) dependant on prey type with conspecifics present (■) or absent (▲).

The probability of little penguins associating with conspecifics was greater when foraging on schooling prey than when on solitary prey (Fig 2a). The number of prey captured per dive was highest for krill, both when hunting alone or with conspecifics, followed by schooling juvenile *Clupeiformes* hunted with conspecifics (Fig 2b). There was little difference for the remainder of prey types regardless of hunting strategy (solitary or with conspecifics). However, despite their numerical abundance, krill comprised the lowest energy gain per dive due to their small size (Fig 2c). In contrast, adult schooling fish (anchovy, sprat) provided the highest energy gain per dive (whether schooling or solitary) followed by juvenile *Clupeiformes* and jellyfish. Interestingly, there was no difference between the energy gained per dive hunting schooling or solitary energy-rich adult fish. More importantly, little penguins hunting schooling anchovy and sprat had greater energy gains per dive when on their own than with conspecifics (Fig 2c).

To investigate whether the time individuals spent foraging on particular prey was influenced by the presence of conspecifics, prey patch residency time (defined as the time between when the first and last prey of a particular type were visible in a patch) was determined. There was no difference found in patch residency time for individuals foraging on schooling anchovy or sandy sprat when foraging alone or with conspecifics *(p*>0.05 in both cases).

## Discussion

Recently, through the use of animal-borne video cameras, it has been possible to gain further insight into the foraging behaviour of numerous penguin species [26–28]. The prey species observed in the present study are largely consistent with previous reports of the diet of little penguins [10], the exception being the large number of Cyaneidae jellyfish (28% of all observed consumed prey items). While the consumption of jellyfish by seabirds has previously been reported, their rapid digestive deterioration means they may often be overlooked in diet studies [29]. In the present study, individuals were observed to consume both whole small jellyfish and the inner portions of larger jellyfish which are the most nutritious components [19].

Due to the video sampling being intermittent, the total number of dives individuals conducted with conspecifics or alone, and in what proportions of these prey were encountered, could not be determined. Consequently, analyses were limited to dives in which prey were encountered. It is possible differences may occur in the rate at which various prey types are detected depending on whether individuals are in association with conspecifics or hunting alone. However, the focus of the present study was to determine if differences existed between individuals hunting alone or with conspecifics in the ability to capture prey once they were detected.

While jellyfish are relatively easy to capture due to their slow mobility, they are generally of low nutritional content [19] and, whereas the estimated energy gain per dive from their consumption was similar to that of juvenile *Clupeiformes* and krill, their longer handling time (and associated dive durations) would likely result in a lower foraging efficiency. Indeed, while approach from below may facilitate accessing the most nutritious components, jellyfish were mostly consumed during the ascent phase of dives to much greater depths where no other prey were encountered. This suggests that individuals were searching for higher quality prey (e.g. fish) but, when unsuccessful, opportunistically consumed jellyfish on ascent.

With the exception of krill and, to a lesser extent, juvenile *Clupeiformes*, little penguins rarely captured more than 1–2 items per dive when hunting schooling prey in association with conspecifics. The relatively low mobility of krill would suggest individuals were not aggregating prey when foraging amongst conspecifics and received minimal benefit from hunting it in the presence of other penguins. Indeed, it was observed that numerous krill were captured with little effort as individuals swam through a swarm. In contrast, juvenile *Clupeiformes* were actively pursued. Contrary to previous assumptions [30], this and other fish species were not captured only from below, with little penguins attacking them as well from above and laterally.

The herding of small prey into tightly aggregated patches for efficient exploitation is observed in numerous predators [31]. In the present study, little penguins were more likely to associate with conspecifics when encountering schooling prey than solitary prey. This is consistent with a predatory strategy of aggregating small prey for efficient exploitation [32] and recent findings suggesting that little penguins spend extended periods at sea foraging with conspecifics [9]. However, for the majority of schooling prey species, individuals did not gain more energy per dive hunting with conspecifics than when alone. Indeed, for anchovy and sprat, energy gain per dive was significantly greater when individuals encountered schools alone. These findings suggest that capture success at prey patches may not necessarily be improved by associating with conspecifics.

In social species where small family groups hunt together cooperatively (e.g. *Panthera leo*), such activities have direct inclusive fitness benefits [4]. In contrast, in non-social species where group foraging occurs, cooperation would deliver no such benefits and the potential for interference competition could influence individual strategies and outcomes of such associations [33]. In the present study, little penguins did not appear to be actively cooperating with coordinated movements and, at times, competed for prey items. In addition, there was no difference in the number of dives made or the amount of time individuals hunted on patches of schooling prey when alone or with conspecifics. While it was not possible to quantify the size of prey schools observed, these results suggest at-sea associations in this species may primarily serve a function other than facilitating the capture of small schooling prey.

Trade-offs between foraging and other factors affecting individual survivorship may impact hunting behaviour. For example, animals could forage as a group to decrease the risk of predation [34, 35]. Predator avoidance strategies have been suggested as a mechanism for group behaviour in some penguin species [27, 36]. Indeed, little penguins aggregate at sea in close proximity to the colony prior to coming ashore, a behaviour commonly assumed to be related to predator avoidance [37]. Individuals in the current study were also seen to raft with conspecifics between foraging bouts which suggests birds may also congregate in groups at sea in order to reduce the likelihood of predation. Alternatively, individuals may associate at sea to increase the likelihood of finding new prey patches by exploiting the abilities of other foragers [38]. Numerous studies [5] have suggested seabirds increase their probability of finding prey by observing conspecific and inter-specific foraging movements (*local enhancement*). Due to the intermittent video sampling, it was not possible in the present study to determine whether individuals increased their frequency of prey patch detection when with conspecifics. However, the majority of associations with conspecifics observed involved surface travel in search of prey. In addition to conspecific associations, individuals in the present study were also observed on some occasions at the surface viewing flying short-tailed shearwaters (*Puffinus tenuirostris*) and subsequently foraging in the same region, such that they may follow them to patches of small schooling fish (S2 Video).

In summary, the results of the present study suggests at-sea group formations in little penguins may serve to increase the detection of small patches of highly mobile prey in a spatially and temporally variable environment rather than facilitate their capture. As little penguins are at risk of being predated on by higher order predators, group foraging may also serve as a strategy in response to predation fear. Whether such behaviour is opportunistic in nature or represents complex strategies with individuals preferentially choosing who to associate with, when and for how long remains to be determined.

## Supporting Information

S1 Appendix Deployment summary of little penguins instrumented with camera, GPS and depth recorder (i). Representative GPS tracks were overlayed with recording periods from Gabo Island (a) and London Bridge (b) (ii).
(DOCX)

S1 Data Prey events and modelling data. (CSV)

S1 Table Results of ANOVA performed on mixed-effects models used to assess the differences in conspecific association, prey capture rate and estimated energy gained per dive. (DOCX)

S1 Video Representative foraging behaviour in little penguins (a). (MP4)

S2 Video Representative foraging behaviour in little penguins (b). (MP4)
