# Supplementary material for: Benefits of Group Foraging Depend on Prey Type in a Small Marine Predator, the Little Penguin
Source: PLoS One. 2015 Dec 16;10(12):e0144297. doi: 10.1371/journal.pone.0144297 (PMC4682954; doi:10.1371/journal.pone.0144297)
Supplement: S1 Appendix — Representative GPS tracks were overlayed with recording periods from Gabo Island (a) and London Bridge (b) (ii). (DOCX) [file pone.0144297.s001.docx]

**S1 Appendix**: Deployment summary of little penguins instrumented with camera, GPS and depth recorder (i). Representative GPS tracks were overlayed with recording periods from Gabo Island (a) and London Bridge (b) (ii).

(i)

| Site | Sex | Total | Body mass (g) | Video data duration (h) | Total trip duration (h) * | Dive depth * |
| --- | --- | --- | --- | --- | --- | --- |
| Gabo Island | M | 6 | 1128 ± 29.3 | 3.7 ± 0.7 | 17.5 ± 0.4 | 7.1 ± 0.1 |
|  | F | 5 | 1038 ± 46.0 | 3.9 ± 0.3 | 17.5 ± 0.1 | 5.7 ± 0.2 |
| London Bridge | M | 5 | 1120 ± 43.9 | 3.1 ± 0.1 | 17.5 ± 0.6 | 12.3 ± 0.2 |
|  | F | 5 | 984 ± 43.0 | 3.6 ± 0.6 | 12.4 ± 2.3 | 5.7 ± 0.1 |
| **n* = 14 due to malfunction of GPS and TDR units | | | | | |  |


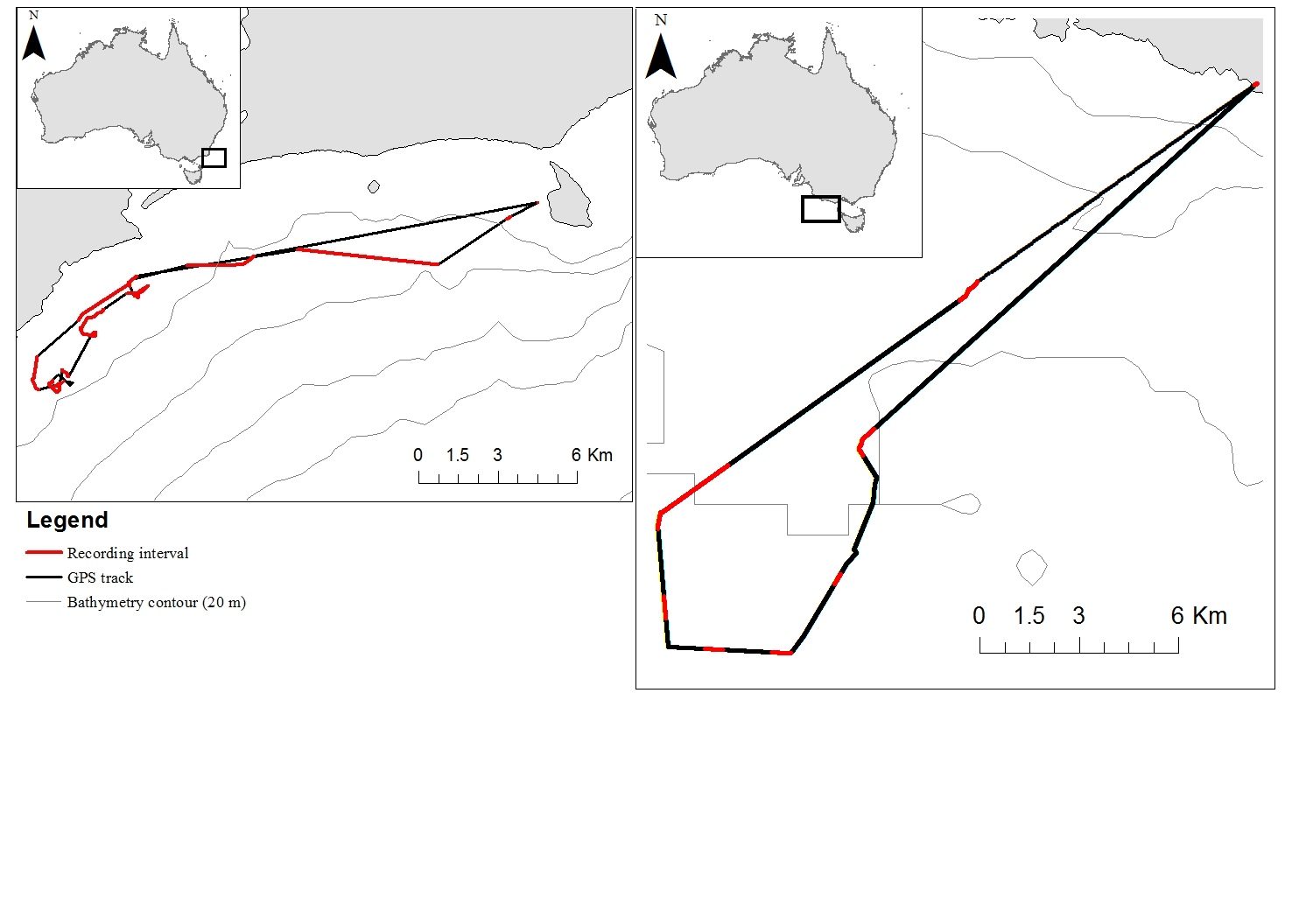


(ii)

(b)

(a)
