# Supplementary material for: Benefits of Group Foraging Depend on Prey Type in a Small Marine Predator, the Little Penguin
Source: PLoS One. 2015 Dec 16;10(12):e0144297. doi: 10.1371/journal.pone.0144297 (PMC4682954; doi:10.1371/journal.pone.0144297)
Supplement: S1 Table — (DOCX) [file pone.0144297.s003.docx]

**S1 Table:** Results of ANOVA performed on mixed-effects models used to assess the differences in conspecific association, prey capture rate and estimated energy gained per dive.

| Response variables | Predictor variables | SSE | MSE | DF1 | DF2 | F | *p* |
| --- | --- | --- | --- | --- | --- | --- | --- |
| Association | Prey Type | 28.045 | 3.5 | 8 |  | 3.51 | <0.05 |
| Average capture per dive | Association |  |  | 1 | 256 | 6.99 | 0.0087 |
|  | Prey Type |  |  | 8 | 256 | 31.61 | <0.0001 |
|  | Association:Prey Type |  |  | 8 | 256 | 7.45 | <0.0001 |
| Gross energy gained per dive | Association |  |  | 1 | 256 | 35.75 | <0.0001 |
|  | Prey type |  |  | 8 | 256 | 82.02 | <0.0001 |
|  | Association:Prey Type |  |  | 8 | 256 | 6.3 | <0.0001 |

SSE: Sum of squared error. MSE: mean squared error

Note: p-values displayed from models that used the *lme4* package (Association and Average capture per dive) have been estimated from the F statistics
